# Gamma frequency transcranial alternating current stimulation over the visual cortex modulates contour integration

**DOI:** 10.3389/fcogn.2026.1715655

**Published:** 2026-05-04

**Authors:** Yosun Yoon, Sang Wook Hong

**Affiliations:** 1 Department of Psychology, Florida Atlantic University, Boca Raton, FL, United States; 2 Stiles-Nicholson Brain Institute, Florida Atlantic University, Boca Raton, FL, United States

**Keywords:** contour integration, early visual cortex, gamma oscillations, perceptual grouping, transcranial alternating current stimulation (tACS)

## Abstract

**Introduction:**

The visual system must integrate spatially fragmented information to perceive coherent object boundaries. Contour integration represents a fundamental process underlying this perceptual organization by linking discrete edge elements into continuous contour. Gamma oscillations have been associated with perceptual grouping processes, from contour integration to object perception; however, their causal contribution to contour integration remains unclear.

**Methods:**

Participants performed a contour detection task in which they identified the orientation of a target contour composed of four collinearly aligned segments embedded among randomly oriented and positioned distractors. To investigate the role of gamma oscillations in contour integration, participants were randomly assigned to one of three groups: gamma-tACS (40 Hz), theta-tACS (7 Hz; active control), or sham stimulation (control condition). Participants completed both pre-stimulation and stimulation sessions. During the stimulation session, the assigned tACS was applied over the early visual cortex.

**Results:**

A mixed-effects linear model analysis revealed a significant session × group interaction, such that contour detection performance improved from the pre-stimulation session to the stimulation session specifically in the gamma-tACS group, whereas no comparable improvement was observed in the sham and theta-tACS groups.

**Discussion:**

These findings provide causal evidence that gamma-frequency stimulation over occipital cortex can facilitate contour integration, suggesting that gamma-band oscillations contribute to the integration of spatially distributed visual elements into coherent contours.

## 1 Introduction

Our daily interactions with the visual world rely on the ability to integrate fragmented visual information into coherent objects. This is because incoming visual input is initially decomposed by the visual system into multiple feature dimensions such as color, shape, size, and motion, which are registered and processed in partially distinct neural pathways (Livingstone and Hubel, 1988). Moreover, neurons in the early visual cortex typically have small receptive fields, and thus, different parts of the same object are represented by multiple neurons when the object extends beyond the receptive field of any single neuron (Roelfsema, 2006). Consequently, to perceive an object as a coherent whole, the fragmented components that compose the object must be integrated into a unified entity. This process, known as perceptual binding or grouping, is essential for object recognition in complex visual environments. Understanding how local visual fragments become combined into global forms is critical for visual perception and object recognition.

Contour integration is an exemplary model for such fundamental processes of perceptual grouping. In contour integration, spatially separated local edges are grouped into a continuous contour based on their geometric properties such as orientation (Field et al., 1993). In a typical contour detection task, participants detect or identify a target contour composed of multiple, segmented units (e.g., Gabor patches) embedded within a background of randomly oriented elements (distractors). Classical accounts emphasize a bottom-up mechanism in which lateral and long-range interactions among orientation-selective neurons facilitate the grouping of collinear or smoothly aligned elements, while suppressing those that disrupt continuity (Ts'o et al., 1986; Hirsch and Gilbert, 1991; Weliky et al., 1995; Polat et al., 1998; Gray and Singer, 1989; Mioche and Singer, 1989; Bosking et al., 1997; Le Bec et al., 2022). This process improves the detectability of a target contour by strengthening signals associated with its global structure while reducing noise from distractor elements. At the same time, contour integration may not be solely mediated by bottom-up mechanisms. A growing body of literature suggests that top-down feedback from higher-order parietal and fronto-occipital networks also contributes critically to contour perception and perceptual organization (Volberg and Greenlee, 2014; Bastos et al., 2015). For example, goal-directed attention regulates distractor suppression and therefore modulates the visibility of a contour within a noisy background (Li et al., 2008; McMains and Kastner, 2011). Thus, contour integration is better understood as the outcome of an interaction between bottom-up grouping of local elements and top-down modulation that shapes which grouped structure ultimately reaches perception. Evidence that lateral occipital regions can drive information flow back toward primary visual cortex during contour grouping is consistent with this view and suggests that recurrent processing is an important component of contour perception (Roelfsema, 2006; Wokke et al., 2013).

This bottom-up/top-down framework maps naturally onto current theories of cortical oscillations. Neural oscillations are increasingly viewed not simply as epiphenomena of perception and cognition but as dynamic mechanisms coordinating communication across distributed cortical networks (Fries, 2015). Within this framework, gamma-band activity has often been associated with feedforward sensory processing and the local binding of stimulus features, whereas alpha-beta rhythms have been linked more strongly to feedback signaling and top-down influences. Supporting this view, on the one hand, a growing body of empirical studies demonstrate that neural oscillations at gamma-frequency range (typically ~30–80-Hz) constitute an important neural mechanism for the integration of segmented visual information to achieve a unified representation of an object (Engel and Singer, 2001; Fries, 2005; Buzsáki and Wang, 2012; Castellano et al., 2014). It has been consistently observed that gamma-band activity is enhanced when observers view coherent objects or grouped stimuli compared to random or scrambled ones. This enhancement suggests a close link between gamma oscillation dynamics and grouping-related perceptual processes (Tallon-Baudry et al., 1996, 1999; von Stein et al., 2000).

On the other hand, more recent work has emphasized the role of beta oscillations in visual processing, particularly within dorsal and parietal systems that support rapid top-down influences on sensory representations (Di Dona and Ronconi, 2023). Pronounced beta-band activity has been consistently observed in the lateral occipital and primary visual cortices during a contour integration task (Volberg et al., 2013; Volberg and Greenlee, 2014). In a related vein, beta-oscillation-mediated top-down influence on contour perception was evident in the regime of visual crowding. Crowding and contour integration seem to be opponent phenomenologically because the former reflects a failure to individuate a target in the presence of neighboring flankers, whereas the latter requires the selective grouping of relevant elements while segregating them from noise. However, these two phenomena reflect how the visual system balances integration and segregation among nearby elements (Chakravarthi and Pelli, 2011). For this reason, crowding and contour integration have often been discussed as related expressions of shared organizational constraints in spatial vision. Importantly, recent studies provide causal evidence that beta-frequency stimulation over parietal regions can modulate crowding performance. Transcranial alternating current stimulation (tACS) in beta-band frequency on parietal cortex has been shown to reduce crowding relative to control conditions (Battaglini et al., 2020), and more recent work has further implicated parietal beta-band activity in resolving crowding (Di Dona et al., 2024). These findings suggest that beta oscillations play a causal role in visual integration/segregation processes, likely through top-down perceptual organization and visuo-spatial selection. By contrast, although gamma activity has long been associated with grouping and feature binding, causal evidence that externally modulating gamma oscillations improves contour integration remains limited.

To bridge this gap, we investigated the putative causal contribution of gamma oscillation to contour integration using tACS. tACS operates by delivering weak sinusoidal electrical currents through electrodes placed on the scalp, thereby entraining neural activity at the stimulated frequencies (Antal and Paulus, 2013; Herrmann et al., 2013; Vossen et al., 2015). tACS can strengthen intrinsic gamma oscillation due to the entrainment and potentially enhance or modulate visual processing that depend on gamma oscillatory activity. We specifically hypothesized that if gamma oscillation at the early visual processing stage plays a critical role in the grouping of discrete elements into coherent contours, then externally induced and strengthened gamma oscillation in the occipital cortex would be expected to facilitate contour integration. We tested this hypothesis by applying 40-Hz tACS while participants performed a contour integration task. Participants were asked to search for a target composed of four linearly aligned Gabor patches among randomly oriented and positioned Gabor distractors and report whether its orientation was −45° or +45°. Based on our working hypothesis, we predicted that participants receiving 40-Hz tACS would show a significant improvement in contour detection performance during stimulation compared to pre-stimulation performance. In contrast, such improvement would not be observed in the Sham control group participants. We specifically employed 40-Hz stimulation, as prior work has shown that perceptual contour completion with illusory figures (e.g., a Kanizsa triangle) elicits robust non-phase-locked gamma activity in the 30–40-Hz range over posterior visual electrodes (Tallon-Baudry et al., 1996). In addition, we included a 7-Hz theta frequency stimulation condition as an active control, motivated by prior evidence that occipital theta tACS does not enhance contour integration relative to sham (Stonkus et al., 2016). Taken together, the present study tests whether gamma-band plays a causal role in contour integrating, complementing prior beta-tACS evidence for top-down contributions to perceptual organization.

## 2 Methods

### 2.1 Participants

A total of 68 adults with normal or corrected-to-normal vision (based on self-report) participated in the study in exchange for course credit. Participants were randomly assigned to one of three groups: a gamma-tACS group, a theta-tACS group, or a sham control group. Participants whose estimated contour integration thresholds were at or near the lower boundary of the stimulus range (24–48 pixels) were excluded from the analysis, as the staircase procedure could not converge on a reliable threshold estimate under these conditions. In such cases, the measured value likely reflected a floor effect rather than a stable estimate of contour integration sensitivity. Based on this criterion (threshold ≤ 24.5 pixels), data from two participants in the gamma-tACS group, one participant in the theta-tACS group, and one participant in the sham group were excluded. Data from the remaining 63 participants (gamma-tACS group: *n* = 21, mean participant age = 20.57, median age = 19, range 18–31, 17 female; theta-tACS group: *n* = 21, mean participant age = 21.38, median age = 20, range 18–34, 17 female; sham group: *n* = 21, mean participant age = 18.57, median age = 18, range 18–20, 16 female) were included in the analysis. All participants confirmed that they had no metal implants in the head, no implanted electronic devices, no history of neurological disorders or head injuries, no claustrophobia, were not pregnant, and were not currently taking psychoactive medication. All the participants in the present study completed a written informed consent form prior to the experiment. The study was approved by the Florida Atlantic University Institutional Review Board.

### 2.2 Apparatus

The stimuli were generated using Psychophysics Toolbox Version 3 (Brainard, 1997) in MATLAB [MATLAB. 9.7.0.1190202 (R2019b), 2018]. They were presented on a 55″ OLED monitor with a refresh rate of 60-Hz, and a resolution of 1920 × 1080 pixels. A chin rest was used to maintain the viewing distance of 200 cm.

### 2.3 Stimuli and procedure

**Figure 1:**
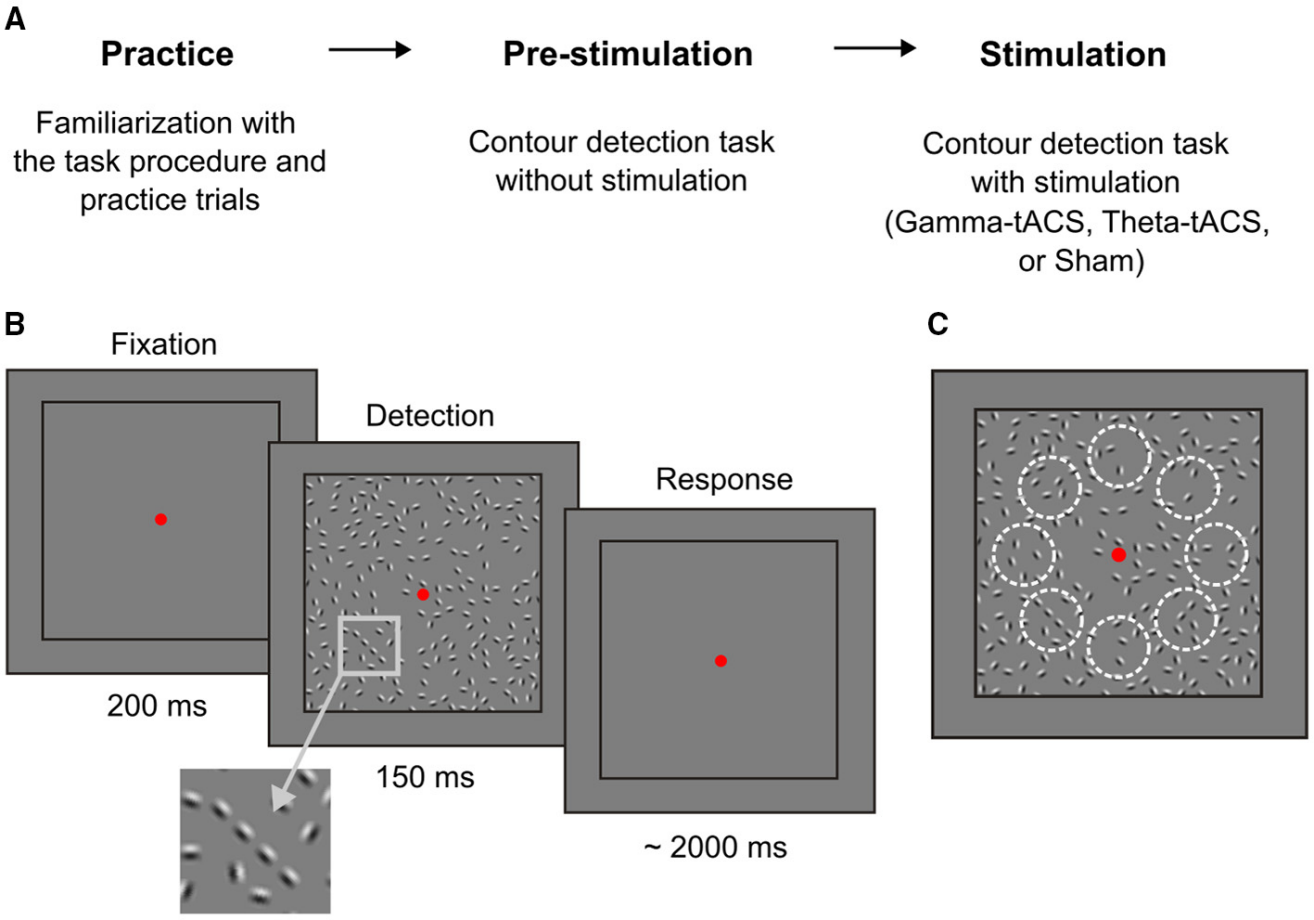
Study design and example of the stimulus sequence within a single trial. **(A)** The experimental procedure consisted of three phases: practice, where participants familiarized themselves with the task procedure; pre-stimulation, where participants performed a contour detection task without stimulation; and Stimulation, where participants completed the same contour detection task while receiving either gamma-tACS (experimental condition), theta-tACS (active control condition), or sham stimulation (control condition). **(B)** After a fixation-only display (200 ms), the target array of Gabor patches appeared for 150 ms before disappearing. Participants had a response window of 2,000 ms. The fixation point, screen, and stimulus sizes are not depicted to scale. **(C)** The target contour appeared at one of eight equally spaced locations along a circular path centered on fixation, positioned at 45° intervals (0°, 45°, 90°, 135°, 180°, 225°, 270°, and 315° polar angles) at a fixed eccentricity. The white dashed circles illustrate the possible target locations and were not shown during the actual experiment.

To investigate whether externally induced gamma activity enhances contour integration, participants completed a contour detection task to assess their sensitivity to target contours among distractors. This task was performed both before stimulation (pre-stimulation phase, serving as a within-subject baseline) and during the stimulation phase, where they received either active or sham stimulation (Figure 1A). The task was adapted from standard contour integration paradigms (Field et al., 1993) and involved identifying the orientation of a target contour embedded in an array of randomly oriented distractors.

The stimuli consisted of Gabor patches with a spatial frequency of 4 cycles/deg, phase = 0°, 100% Michelson contrast, and a Gaussian envelope with a sigma of approximately 0.13°. Each patch subtended 0.43° of visual angle and was arranged within an 11.42° × 11.42° visual field (Figure 1B). Each trial contained a target contour, composed of four aligned Gabor patches forming a straight line oriented −45° or +45°, embedded among distractors that were randomly oriented. The target contour could appear in one of eight possible locations, evenly spaced along an imaginary circle centered on fixation. These locations were equidistant from the center and arranged at 45° intervals, forming a circular array at fixed eccentricity (i.e., 0°, 45°, 90°, 135°, 180°, 225°, 270°, and 315° polar angles; Figure 1C). The stimulus was presented for 150 ms to prevent eye movement from directing to the possible target location. During the 2,000 ms response window, participants were required to report the perceived orientation of the target as quickly and accurately as possible by pressing the corresponding key: the left arrow key for a −45° target and the right arrow key for a +45° target. Participants were instructed to maintain strict fixation on the red dot at the center of the screen for the duration of the task.

### 2.4 Sensitivity measurement of contour integration

A two-down, one-up staircase procedure was implemented to determine the participant-specific threshold of element spacing for contour integration. Spacing between elements is known to be a critical determinant for contour integration (Field et al., 1993; Kovács and Julesz, 1993; Polat and Sagi, 1994). The staircase method is commonly used in psychophysical experiments to estimate perceptual thresholds by dynamically adjusting stimulus difficulty based on participant responses (Levitt, 1971). At the beginning of each session, the initial spacing between elements in the target contour was randomly assigned within a predefined range (24–48 pixels; corresponding to 0.44°–0.87° of visual angle). The staircase procedure was applied separately to each of the eight possible target locations (Figure 1C). For each trial, participants viewed a stimulus display consisting of an array of Gabor patches and were required to identify the orientation of the target contour. Responses were recorded using designated response keys: the left arrow key for −45° orientation and the right arrow key for +45° orientation.

The staircase procedure followed an adaptive approach where the spacing between contour elements was modified based on participant accuracy. This adaptive procedure converges to approximately 70.7% correct performance, providing an estimate of the participant-specific contour integration threshold (Levitt, 1971). If a participant responded correctly in two consecutive trials, the spacing increased, making the task more challenging. Conversely, if a participant made an incorrect response, the spacing decreased, making the task easier. The step size of these adjustments followed a predefined sequence (step sizes: 5, 5, 3, 3, 2, 2, 1 pixels; equivalent to 0.089°, 0.089°, 0.054°, 0.054°, 0.036°, 0.036°, and 0.018° of visual angle) to allow for finer adjustments as the staircase progressed. Reversals, defined as a change in the direction of staircase movement (from increasing to decreasing spacing or vice versa), were tracked for each target location. The staircase procedure for a given location was terminated either after five reversals were recorded or once 50 trials had been completed for that location. Because the staircase procedure adapted dynamically to participant responses, the total number of trials per session varied slightly depending on how quickly the staircase converged. Across participants, the mean number of completed trials per session was 259.8 (SD = 36.6; range = 205–399). The mean number of reversals per staircase was 4.74 (SD = 0.96), indicating reliable convergence of the adaptive procedure.

Threshold estimation was based on the average spacing value from the last five reversals for each target location. These values were then aggregated across locations to compute the final contour integration threshold for each participant. This procedure ensured that the final threshold represented a stable estimate of contour detectability while minimizing potential learning effects or response biases.

### 2.5 Transcranial alternating current stimulation

**Figure 2:**
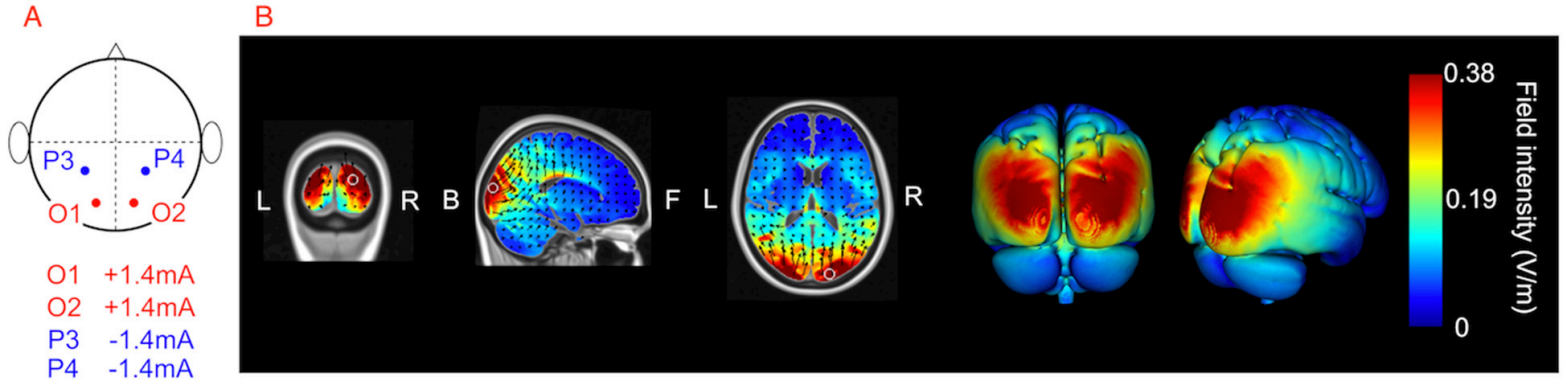
Electrode montage and electric field distribution of HD-tACS. **(A)** Schematic of electrode placement and current configuration, with two anodes (O1, O2; +1.4 mA each) and two cathodes (P3, P4; −1.4 mA each). **(B)** Electric field distribution across axial, sagittal, and coronal slices simulated with the high-definition transcranial alternating current stimulation (HD-tACS) montage. 3D surface projections show that the strongest field intensity was concentrated over the occipital cortex. The color scale indicates field intensity (V/m).

To apply 40-Hz tACS over the early visual cortex, a high-definition tACS system (Soterix M × N-5 High Definition Transcranial Electrical Current Stimulator, Model 9002A, Soterix Medical, New York, NY) was used. Five sintered Ag/AgCl electrodes were attached to high-definition plastic holders, filled with conductive gel, and embedded in the Biosemi EEG cap. To achieve a model-based electrical stimulation, electrode montages (Figure 2) for the occipital regions were created using the Soterix HD-Explore software (version 4.1, Soterix Medical, New York, NY).

For the gamma-tACS group, stimulation at 40-Hz was delivered using electrodes placed over the left and right visual cortex (O1 and O2) and parietal sites (P3 and P4). For the theta-tACS group, the same montage was used, but stimulation was applied at 7-Hz. The electrode locations and current intensities for both conditions are shown in Figure 2. Although the montage included P3 and P4, electric field modeling indicated that the strongest stimulation was concentrated over the occipital visual cortex. During task performance, a bipolar sinusoidal alternating current (40 or 7-Hz, 1.4 mA peak-to-peak) was applied for approximately 15 min. The sham control group followed the same procedure as the tACS group, but the electrical current was gradually ramped up for 30 s right after starting the stimulation and ramped down for 30 s to 0 mA at the beginning and the end of stimulation, simulating the tingling sensation that subjects typically experience. The study was conducted in a single-blind manner, such that participants were unaware of their stimulation condition. A post-experiment safety questionnaire (Poreisz et al., 2007) and a visual analog scale (Gandiga et al., 2006), organized and provided by Reinhart et al. (2017), were administered to assess participants' experiences of potential side effects—such as headache, concentration difficulties, mood changes, visual changes, fatigue, and sensations under the electrodes—as well as to evaluate overall discomfort and levels of attention and fatigue. Scores on these ratings did not significantly differ between the three groups (all *ps* > 0.09).

### 2.6 Statistical analysis

An a priori power analysis was conducted to determine the required sample size for detecting a statistically significant interaction effect in a 2 × 3 mixed-design ANOVA (between-subjects factor: stimulation group with three levels—gamma, theta, and sham; within-subjects factor: session with two levels—pre-stimulation, stimulation) using G^*^Power 3.1 (Faul et al., 2007). Ideally, the most appropriate reference would be a previously reported interaction effect; however, to our knowledge, no directly comparable estimates were available in the literature. We therefore based our a priori effect size on the most conceptually relevant prior tACS study of perceptual integration (Stonkus et al., 2016), which employed a within-subject design with five stimulation conditions (including sham) and reported a main effect of condition, *F*(4, 80) = 2.48, *p* = 0.05, corresponding to partial η^2^ ≈ 0.11 (Cohen's *f* ≈ 0.35); the in-phase vs. sham contrast was *t*_(20)_ = 2.92 (partial η^2^ ≈ 0.30). Although this represents a main effect rather than an interaction, it provides the closest empirical benchmark for the expected magnitude of stimulation-related differences in perceptual integration. Because effect sizes from within-subject designs can be inflated relative to between-subject designs due to reduced error variance, we adopted a smaller a priori partial η^2^ = 0.06 (Cohen's *f* ≈ 0.25)—i.e., conservative relative to Stonkus—while noting that true tACS effects can be smaller.

In G^*^Power, we selected *F* tests → ANOVA: Repeated measures, within–between interaction, with α = 0.05, power (1–β) = 0.80, three groups, two measurements, correlation among repeated measures set to 0.5, and ε = 1. Under these parameters, the required total sample size was estimated as *N* = 42 (14 per group). To increase statistical robustness and account for potential attrition, we targeted a larger sample of *N* = 63 (21 per group), which also provided sufficient power for the mixed-effects analyses used in the final statistical model.

A sensitivity analysis with *N* = 63 indicated that the minimal detectable session × group interaction effect corresponded to approximately *f* = 0.23 (η^2^*p* ≈ 0.050) at ρ = 0.3, *f* = 0.20 (η^2^*p* ≈ 0.038) at ρ = 0.5, and *f* = 0.16 (η^2^*p* ≈ 0.025) at ρ = 0.7. These values confirm that our design had adequate power to detect small-to-moderate interaction effects in the expected range reported by prior tACS studies.

All statistical analyses were conducted in Python (version 3.12.4). The Shapiro–Wilk normality tests were performed using the scipy.stats module from SciPy (version 1.16.3). Linear mixed-effects models and Tukey's HSD *post-hoc* comparisons were implemented using the statsmodels package (0.14.6). Supporting libraries included NumPy (version 2.3.5) and pandas (version 2.3.3). Before conducting the primary analyses, we examined distributional assumptions within each cell of the 3 (group: gamma, theta, sham) × 2 (session: pre-stimulation, stimulation) design. Shapiro–Wilk tests indicated no significant deviations from normality in any condition (all *ps* > 0.05), and inspection of Q–Q plots supported the same conclusion. Because the within-subject factor had only two levels, the assumption of sphericity was inherently satisfied (ε = 1). Although the power analysis was based on the mixed-design ANOVA framework implemented in G^*^Power, the confirmatory analyses were conducted using linear mixed-effects models because they provide greater flexibility for modeling repeated-measures data and subject-level variability. The primary confirmatory analysis tested the session × stimulation condition interaction using a linear mixed-effects model. Session, stimulation condition, and their interaction were included as fixed effects, with subject included as a random intercept. The model was specified as: threshold ~ session × stimulation condition + (1 | subject) with subject included as a random intercept. Categorical predictors were coded using treatment (dummy) coding, with the gamma-tACS condition and the pre-stimulation session serving as the reference levels.

## 3 Results

### 3.1 Baseline comparisons

At baseline (pre-stimulation), there were no significant differences among the three groups. A one-way ANOVA showed no main effect of stimulation condition on threshold, *F*(2, 60) = 0.36, *p* = 0.702, and η^2^*p* < 0.012. Thus, the three groups started from comparable baseline performance.

### 3.2 Mixed-effects linear model

To account for subject-level variability and the repeated-measures structure of the data, we analyzed the effects of session and stimulation condition using a linear mixed-effects model with subjects included as a random intercept. The model included session (pre-stimulation vs. stimulation), stimulation condition (gamma-tACS, theta-tACS, and sham), and their interaction as fixed effects. The analysis revealed a significant main effect of session (β = 4.00, SE = 0.43, *z* = 9.26, and *p* < 0.001), indicating that the spacing threshold increased from the pre-stimulation to the stimulation session overall. The main effect of stimulation condition was not significant for either theta-tACS (β = 1.15, SE = 1.37, *z* = 0.84, and *p* = 0.402) or sham (β = 0.56, SE = 1.37, *z* = 0.41, and *p* = 0.683), relative to the gamma-tACS reference level.

**Figure 3:**
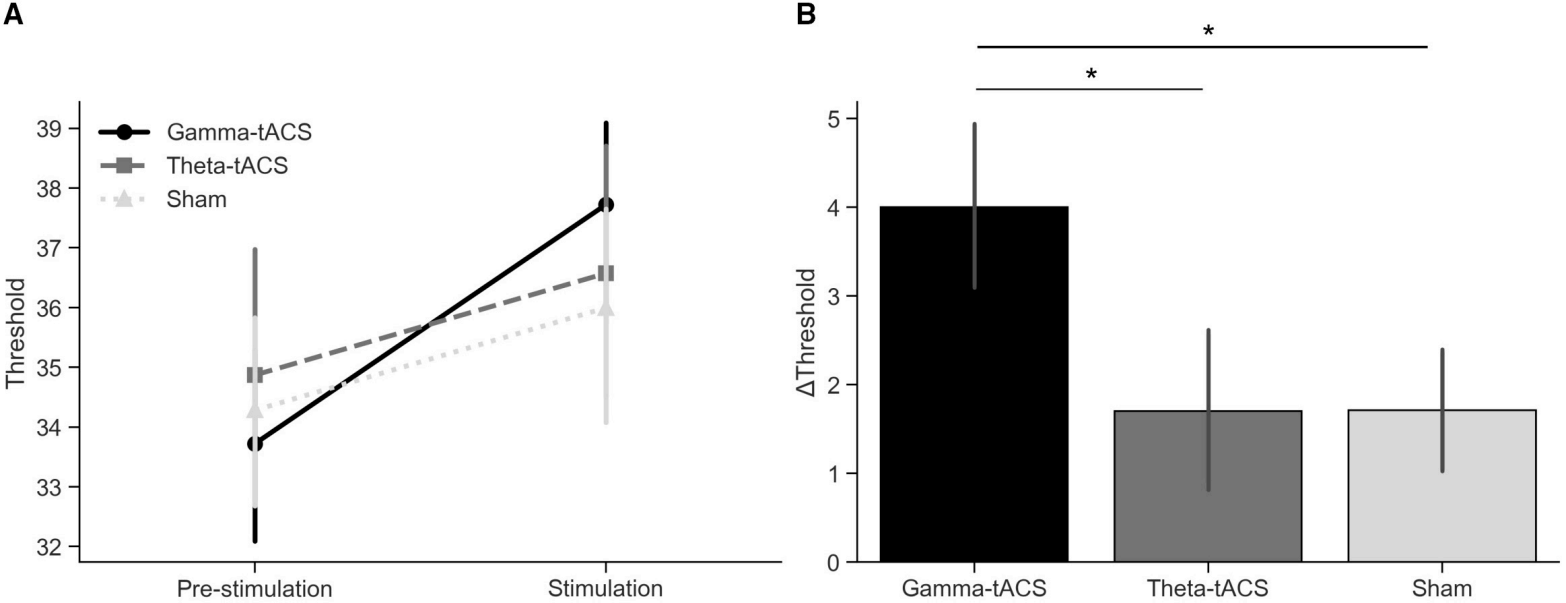
Effect of tACS on target contour detectability. Measured thresholds, defined as the minimum spacing between contour elements required to detect the target, across stimulation conditions. **(A)** Interaction plot showing mean threshold values at pre-stimulation and stimulation sessions for each group. Symbols and line types indicate stimulation group (Gamma-tACS, black circles/solid line; Theta-tACS, dark-gray squares/dashed line; Sham, light-gray triangles/dotted line). **(B)** Bar graph of change scores (stimulation minus pre-stimulation) by group. Error bars represent 95% confidence intervals. Horizontal bars with asterisks indicate significant planned contrasts (*p* < 0.05).

**Figure 4:**
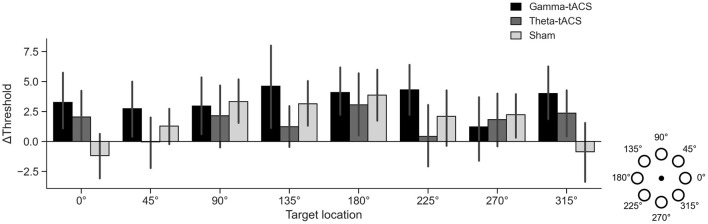
Changes in contour integration threshold across target locations. Mean changes in threshold (stimulation–pre-stimulation) are shown for each of the eight target locations and stimulation conditions (gamma-tACS, theta-tACS, and sham). Error bars represent ±1 SEM across participants. The overall pattern of threshold changes was broadly consistent across spatial locations, with no systematic location-specific effects.

Critically, the interaction between session and stimulation condition was significant. Compared with the gamma-tACS condition, the change from pre-stimulation to stimulation was significantly smaller in the theta-tACS condition (β = −2.30, SE = 0.61, *z* = −3.76, and *p* < 0.001) and in the sham condition (β = −2.29, SE = 0.61, *z* = −3.74, and *p* < 0.001). These results indicate that the increase in threshold from pre-stimulation to stimulation was significantly larger in the gamma-tACS group than in the theta-tACS or sham groups (Figure 3). Follow-up pairwise comparisons using Tukey's HSD test confirmed that thresholds significantly increased from pre-stimulation to stimulation in the gamma-tACS condition (mean difference = 4.00, *p* = 0.047, and 95% CI [0.03, 7.97]). No other pairwise comparisons were significant (all *ps* > 0.12). Threshold changes across the eight target locations are illustrated in Figure 4. The overall pattern of results was broadly consistent across locations, with no systematic spatial bias that could account for the observed stimulation effects.

## 4 Discussion

In the current study, we investigated whether externally applied gamma-band tACS in occipital cortex modulates contour integration. We found that participants receiving 40-Hz tACS showed a significant improvement in the contour detection task during stimulation compared to the pre-stimulation baseline, whereas theta-tACS and sham groups did not exhibit comparable improvement. This frequency-specific improvement was supported by the linear mixed-effect model analysis revealing a significant session × group interaction, suggesting that the effect is unlikely to be explained solely by general arousal or practice effects. Our results provide behavioral evidence that externally enhanced gamma-band activity over occipital cortex can facilitate integration of spatially fragmented contour elements.

Our results support the hypothesis that gamma-band oscillations contribute to the neural mechanisms of perceptual grouping. Previous studies have associated gamma-band synchronization with the binding of spatially distributed visual features and therefore with coherent object perception. However, most of this evidence has been correlational, relying on electrophysiological recordings in animals (Eckhorn et al., 1988; Gray and Singer, 1989; König et al., 1995; Livingstone, 1996) and in humans (Tallon-Baudry et al., 1996). The current study extends this literature by demonstrating that external modulation of gamma-band activity can result in changes in contour integration performance. The findings thus suggest that gamma-band synchronization may play a causal role in neural mechanisms underlying both perceptual grouping and contour integration.

In the current study, 40-Hz tACS applied over the occipital regions was expected to entrain and/or enhance oscillatory brain activity at the applied frequency, thereby facilitating early-stage processing of local contour elements and their orientation coherence. However, our findings should be interpreted within the broader framework of distributed oscillatory dynamics supporting visual object perception. Contour integration is unlikely to rely solely on local gamma-band activity in early visual cortex. Local oscillatory activity in the visual cortex is thought to interact with larger-scale neural circuits to achieve global percepts. For example, long-range gamma-band synchronization across the fronto-parietal regions significantly predicted successful contour detection performance (Castellano et al., 2014). Other research consistently supports this distributed oscillatory network framework and has demonstrated the involvement of oscillatory activity in other frequency bands. For instance, alpha-band (8–12-Hz) oscillations have been typically associated with attentional regulation of contour processing (Kinsey et al., 2009). Similarly, beta-band activity has been linked to top-down influences during perceptual grouping (Tarokh, 2009; Volberg and Greenlee, 2014). Taken together, these observations highlight that contour integration likely emerges from the interplay of multiple oscillatory mechanisms across distributed cortical networks. Within this framework, the improved contour detection observed in the current study highlights the complementary contribution of gamma-band activity, which may support the initial grouping of contour elements that subsequently interacts with higher-order processes of perceptual integration.

One important consideration when interpreting the present findings concerns the nature of the stimuli used in the contour detection task. In the current study, the target contours consisted of straight linear arrangements of Gabor patches oriented at ±45°. Although this design allowed for precise control of contour orientation and alignment, many contour integration studies employ more complex configurations, including curved contours or closed shapes (Field et al., 1993; Hess et al., 2001; Dakin and Baruch, 2009; Castellano et al., 2014; Volberg and Greenlee, 2014). Such stimuli engage additional geometric constraints such as circularity and curvature continuity, which may recruit partially distinct perceptual mechanisms. Therefore, while the present results demonstrate that gamma-frequency stimulation can facilitate detection of simple linear contours, future studies will be needed to determine whether similar effects generalize to more complex contour structures. Extending the paradigm to include curved or closed contours would help clarify the broader applicability of gamma-mediated facilitation in perceptual grouping.

Several methodological limitations should also be acknowledged. First, the use of a fixed 40-Hz tACS across all participants, rather than tailoring the stimulation to individual peak gamma frequency, may limit the effectiveness of gamma-band tACS. A growing body of research emphasizes the importance of applying individualized peak frequencies to achieve effective neural entrainment in brain stimulation. In addition, considering that previously reported individual gamma frequencies have been shown to vary depending on the type of visual processing, brain states, and cognitive processes involved (see Mockevičius et al., 2023 for review), optimizing individual gamma frequency would be beneficial in future studies. Second, the current study did not include direct neurophysiological measurement of oscillatory activity before and after stimulation. Consequently, it remains unclear whether the observed behavioral improvements were directly mediated by measurable changes in gamma-band activity in occipital cortex. Combining tACS with EEG/MEG in future work would provide valuable insights into the neural mechanisms underlying the observed behavioral effect.

Despite these limitations, the current study demonstrated that externally modulating gamma-band oscillations can enhance contour integration performance. By providing causal evidence linking gamma-band oscillations to perceptual grouping, this study contributes to a growing body of work suggesting that neural oscillations play a crucial role in shaping visual object perception. More broadly, this finding highlights the potential of frequency-specific brain stimulation as a tool to probe neural mechanisms of perceptual organization and to modulate visual processing in a targeted manner.

## Data Availability

The datasets presented in this study can be found in online repositories. The names of the repository/repositories and accession number(s) can be found below: https://osf.io/5qp8w/.
